# Design of the Advance Directives Cohort: a study of end-of-life decision-making focusing on Advance Directives

**DOI:** 10.1186/1471-2458-10-166

**Published:** 2010-03-26

**Authors:** Matthijs PS van Wijmen, Mette L Rurup, H Roeline W Pasman, Pam J Kaspers, Bregje D Onwuteaka-Philipsen

**Affiliations:** 1 Department Public and Occupational Health, EMGO Institute for Health and Care Research, VU University Medical Center, van der Boechorststraat 7, Amsterdam, 1081 BT, the Netherlands

## Abstract

**Background:**

ADs are documents in which one can state one's preferences concerning end-of-life care, aimed at making someone's wishes known in situations where he/she is not able to do so in another manner. There is still a lot unclear about ADs. We designed a study aimed at investigating the whole process from the formulating of an AD to its actual use at the end of life.

**Methods/Design:**

The study has mixed methods: it's longitudinal, consisting of a quantitative cohort-study which provides a framework for predominantly qualitative sub-studies. The members of the cohort are persons owning an AD, recruited through two Dutch associations who provide the most common standard ADs in the Netherlands, the NVVE (Right to Die-NL), of which 5561 members participate, and the NPV (Dutch Patient Organisation), of which 1263 members participate. Both groups were compared to a sample of the Dutch general public. NVVE-respondents are more often single, higher educated and non-religious, while amongst NPV-respondents there are more Protestants compared to the Dutch public. They are sent a questionnaire every 1,5 year with a follow-up of at least 7,5 years. The response rate after the second round was 88% respectively 90% for the NVVE and NPV. Participants were asked if we were allowed to approach close-ones after their possible death in the future. In this way we can get insight in the actual use of ADs at the end of life, also by comparing our data to that from the Longitudinal Aging Study Amsterdam, whose respondents generally do not have an AD.

**Discussion:**

The cohort is representative for people with an AD as is required to study the main research questions. The longitudinal nature of the study as well as the use of qualitative methods makes it has a broad scope, focusing on the whole course of decision-making involving ADs. It is possible to compare the end of life between patients with and without an AD with the use of data from another cohort.

## Background

Medicine has made a lot of progress the last ages, with an exponential development in the 20th century. As a result there are answers to problems that remained unsolved in the past. Life-expectancy has grown, at least in the Western world. But besides solving problems, this also created new ones. With its expansion, medicine increasingly interfered in living and, even more so, dying. Where there used to be no choice, nowadays difficult decisions have to be made. Patients are alive in situations where they otherwise would have passed away, which creates dilemmas where length and quality of life sometimes seem to be competing with each other. For instance, the decision to start with artificial feeding in the advanced stage of a critical illness might enable a patient to live longer while at the same time it might lengthen the period of discomfort that the disease will cause. These choices are personal ones, depending on what an individual considers to be the most valuable in living and dying.

Personal preferences can be put down in an advance directive (AD), a written statement concerning possible end-of-life decisions to be made in the future. This document is aimed at stating someone's wishes when he/she is in the situation that he/she is not capable to do so verbally or to emphasize uttered present wishes, which are reflected by the AD. It can either make statements about receiving or refusing certain treatments at the end of life, or appoint a person who will take decisions for the patient in question - a healthcare proxy.

ADs might become increasingly important in the process of decision-making at the end of life. Yet, there is still a lot unclear about ADs. At the moment you could say this has created a controversy about the usefulness of ADs. An argument made in favor of ADs is that the documents or the choices made by proxies reflect patient's wishes and in that way enhance the quality of living and dying at the end of life [1-3]. On the other hand there are doubts if a person is able to make a sound choice about possible end-of-life issues in the future [4,5] or that an appointed proxy is able to take decisions according to another person's preferences [6]. This contributes to the more skeptical sound that is also heard concerning ADs [7-9].

Whether ADs work as they are supposed to or not, they exist and they are used. Especially about the use of ADs, there is still a lot unclear, particularly while it is difficult doing research on this matter because it brings along several ethical and practical issues. The study described here, was designed in order to get a better insight into how decisions are made at the end of life focusing on ADs. It takes place in the Netherlands.

This article focuses on how this study was set up in order to investigate the broad spectrum of research questions related to the whole process ranging from formulating an AD, possibly as a healthy individual, to the eventual use of an AD in case of serious illness (table 1). The design will be explained as well as the recruitment, measurements and sub-studies. Finally a description of the respondent groups in relation to the general Dutch public will be given.

**Table 1:** Research Questions

Subject	Research Questions
Formulating an AD	What are people's preferences at the end of their lives? What are reasons to formulate (or not formulate) an AD?

Having an AD	Do the preferences concerning a person's end of life change over time and does he/she modify his/her AD accordingly if they do? What are the expectations concerning ADs and end-of-life decision-making among people who have an AD and do they discuss this with their physician or others?

The Use of ADs	Are the directions in ADs followed at the end of life in the situations where they relate to? If they are followed, what are the reasons therefore? If they are not followed, how do they influence the end of life of the person in question? What is the role of physicians and family or close-ones in this process?

## Methods/Design

### Design cohort study of people with an advance directive

**Figure 1:**
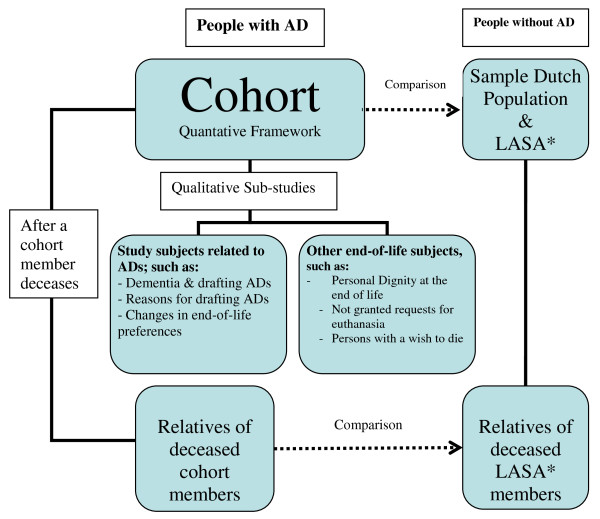
**Scheme of the design of the study**. * LASA = Longitudinal Aging Study Amsterdam.

Mixed methods are used. Central in this study is a prospective quantative cohort-study (fig. 1). The studied population consists of people who have formulated one of the common types of standard ADs in the Netherlands. For the entire cohort data are collected by means of a written questionnaire every one and a half year. The follow-up will be at least seven and a half years.

The cohort provides a quantitative framework from which it is possible to select subgroups for predominantly qualitative sub-studies. An important added value of qualitative studies is to get better insight in certain mechanisms, for instance why end-of-life preferences change. Furthermore it is possible to identify other factors influential on choices at the end of life than the ones already identified by the quantitative study, because of the open view by which qualitative research is characterized.

The data generated by the cohort can be compared with the data from other studies like a sample of the general Dutch population and the Longitudinal Aging Study Amsterdam (LASA). Both will be discussed later in this article, LASA in connection with the study on the use of ADs at the end of life for which relatives of deceased respondents are approached.

### Recruitment

As to include as much people possible who have an AD, we asked the help from the two associations that provide the most common types of standard ADs in the Netherlands. The 'Right to Die-NL' (NVVE in Dutch) is a society that is engaged in the supposed right of an individual to have extensive control over the last phase of his/her life, thus broader than euthanasia alone. They provide three types of standard ADs.

The first, a refusal of treatment document, is an AD where a person states that he/she doesn't wish to receive life-prolonging treatment in the case where his/her quality of life is below a for him/her acceptable level and there is no chance that it will improve. The second is the advance euthanasia directive (AED). Euthanasia is allowed by law in the Netherlands, making it a subject of decision-making at the end of life. The AED is a document, where a person declares that he wants to receive euthanasia in situations where he suffers unbearably and there is no prospect of recovery. These situations can be specified in the AD. AEDs are specifically mentioned as a legitimate request for euthanasia in the Euthanasia Act, which allows - but does not require - a physician to grant a patient's wish to end his/her life in case other requirements are met as well.

Finally there is the AD where someone appoints a person who will take (medical) decisions for him when he is no longer able to - a healthcare proxy.

In 2005 90.637 members of the NVVE had requested a standard AD. An estimate of seventy-five percent of them actually completed the AD, but the exact figure is not known.

The other association, the Dutch Patient Association (NPV in Dutch), a Christian orientated patient association, provides the second most common standard AD in the Netherlands. This is a so-called wish-to-live statement, where a person declares that he/she wants to receive proper care, meaning no excessive, medically useless treatments at the end of his life but also no actions with the purpose of actively terminating his life. In this way he/she expresses his/her wish to die in a dignified and respectful manner. The NPV, unlike the NVVE, has on file which members actually completed an AD. In 2005, 5812 members of the NPV had a wish-to-live statement.

A random sample of people was taken from the membership files of both associations. This sample consisted of people who at some point in the past had requested one of the standard AD's of the NVVE or had completed a wish-to-live statement of the NPV. They were sent questionnaires which also contained a question about if they would be willing to participate in an interview if selected for a (qualitative) sub-study. If selected, the specific participant got a request to give written consent before being interviewed.

In addition they were asked if they were willing to give the name and contact information of two relatives. In the event of their death or incompetence, these relatives could be approached some time afterwards in order to learn more about the circumstances at the end of life, the decision-making involved and the role the AD played in all of this. Off course the relatives are first informed about our study and asked to give written consent before participating.

In case a respondent wasn't able to fill in the questionnaire by him/herself because of physical constraints for instance, we also presented the possibility to complete it by means of an interview by phone.

It is important that the cohort is large enough to contain a sufficient number of people whose (health) situation is changing and go through their last phase of life. Since no relevant data are available, it was not possible to make a power calculation. It was therefore decided to form a large cohort. We now know that between the start of the study and the second time participants were sent a questionnaire after one and a half year in total 276 of them had passed away. This is a sign that over the years of follow-up the cohort will produce enough data on changes in health status and different situations at the end of life.

**Figure 2:**
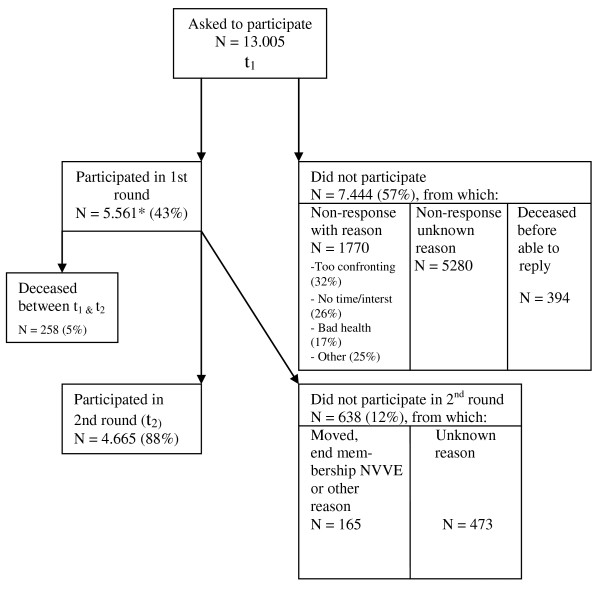
**Flowchart of recruitment and response of members from the NVVE**. * This number consist of people who actually had drawn up an AD and the ones who had only requested one from the NVVE, but had not yet completed it (N = 1.059).

From the NVVE 13.005 members were asked to participate in the longitudinal study, of whom 5.561 (43%) actually did participate (fig. 2). The people who gave a reason why they did not participate (N = 1770), most often said they found the subject too confronting (32%). Other grounds often heard were that people had no time or interest (26%) or too bad health (17%). When the second questionnaire was sent one and a half year later, the response rate was 88% (N = 4.665). The people who had deceased in the intermediate period (N = 258, 5% from the 5.561 original participants), are not included in this calculation. For the majority of the 12% that did not participate in the second round, the reason why was unknown.

**Figure 3:**
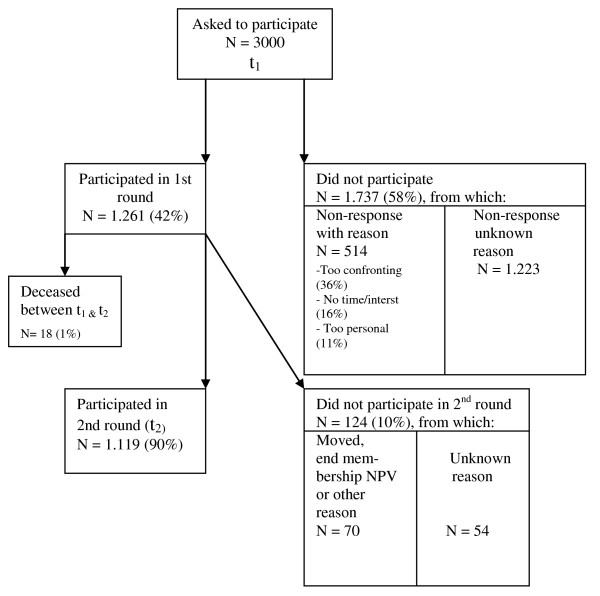
**Flowchart of recruitment and response of members from the NPV**.

From the NPV 3000 members were asked to participate, from which 1263 (42%) actually agreed to do so (fig. 3). From the 58% (N = 1.737) that did not want to take part, the same two reasons as with the NVVE were heard most often as to why this was the case: the subject was too confronting (36%) or that they had no time or interest (16%). The third motivation heard most frequently differs from the NVVE: the subject was too personal for 11% of the members of the NPV who did not want to participate.

When they were sent a questionnaire for the second time, 90% (N = 1.119) from the original participants responded. Again, the 18 persons who had deceased in the meanwhile were not included in the calculation of this response rate.

Although these figures are still preliminary, we can also say something about the response rates of the third round 3 years after the start of the study. For the NVVE the response rate was 79% (N = 4368) and for the NPV 83% (N = 1.043) (both rates based on the number of original participants). At least a fourth, fifth and sixth round will follow in the next years.

### Ethical approval and confidentiality

The Medical Ethics Review Committee of the VU University Medical Center approved the study. Anonymity of the participants was guaranteed where possible. The questionnaires were sent by the two associations involved using their membership files. They were returned to the researchers using only the respondent number. That way the researchers did not get to know the respondent's name and address. The only exceptions were subjects who had agreed to give up their anonymity, should they be selected for one of the qualitative sub-studies.

### Measurements

In order to answer the questions posed by the objectives of this study, an extensive questionnaire was drawn up for the first measurement. It consists of five sections: (1) personal characteristics, (2) personality, (3) health status and quality of life, (4) wishes concerning the end-of-life and (5) ADs. A participant's personality was evaluated using part of the Neurotics-Extroversion-Openness Five Factor Inventory (NEO-FFI), a questionnaire to test personality. In the section about health status and quality of life, the EQ-5D was taken up. This is a standardized instrument to measure health status.

The questionnaires of the follow-up are focused on changes in health status, life-events, quality of life, wishes concerning the end of life and ADs. There is a possibility to add questions relevant to other studies in a follow-up measure. The first follow-up questionnaire for instance included the PDI (ref chochinov Dignity revisited) for use in a study on personal dignity at the end of life.

### Nested sub-studies

The data originating from the questionnaires could also be used to select groups for the qualitative sub-studies, which consist of in-depth interviews. These sub-studies focus on people with dementia, people with a wish to die, denied requests for euthanasia, recently drafted AD's and dignity in the light of specific diseases (like dementia or cancer).

### Study on the use of ADs at the end of life

As to investigate how the process of decision-making at the end of life took place and what role the AD had played, respondents were asked in the questionnaires if the researchers were allowed to approach family or close-ones in case the respondent would pass away during follow up. If family members or close-ones agreed to cooperate with the study after a specific respondent had deceased, they were sent a questionnaire focused on the last phase in the life of the departed.

To get a complete image of the role ADs play at the end of life, we wanted to evaluate both situations in which there was an AD present and in which there was not. We had the possibility to collaborate with the Longitudinal Aging Study Amsterdam (LASA). This is a study focusing on several aspects of aging. Participants, who are representative for the older Dutch population, are interviewed every three years. Besides that parts of our questionnaire were presented to participants in LASA, their family or close-ones were approached in the same way after they had died as with the members of our cohort, so that it was possible to get a look into the process of end-of-life decision-making without an AD.

### Comparison with Dutch public

Although it was not essential in order to be able to answer the main research questions formulated in table 1, we wanted to compare the data from the cohort with the general Dutch population as to look where people with an AD stand in relation to the Dutch population when it comes to background characteristics and views on end-of-life issues. In order to do this parts of the questionnaire used for the Advance Directives cohort were sent to an established sample of the Dutch public: the Consumers' panel for Health services of the Netherlands Institute for Health Services Research (in Dutch: NIVEL). This sample is designed to be representative of the population of the Netherlands of 20 years and older. From the original sample of 1621 persons, 1402 people returned the questionnaire, which makes a response rate of 86%. The respondents were representative for the Dutch population for age, but men were somewhat underrepresented (44% versus 49%), as were first and second generation migrants (7% versus 19% in the general Dutch population in 2005 according to Statistics Netherlands).

Table 2 gives an overview of background characteristics from the respondents from the NVVE, NPV and the Consumers' panel in order to get an outline of the differences between the groups. We split the respondents in a group with the age below 65 years and one above, because age is a possible confounder, for instance regarding marital status (more widowed persons in older age categories). This may lead to a selection bias when it comes to the NVVE- and NPV-respondents because they are on average older compared to the Consumers panel (respectively 67.8 (95% CI 67.5-68.1) and 59.6 (58.6-60.6) versus 50.6 (49.9-51.4)). It can be noticed that both the respondents from the NVVE and NPV differ from the general Dutch population on several other points as well.

**Table 2:** Background characteristics of members of the cohort (NVVE and NPV) and a sample of the Dutch population in 2005 (rounded percentages with 95% confidence intervals).

	**Members of the NVVE (n = 5561)**		**Members of the NPV** **(n = 1261)**		**Sample of the Dutch population (n = 1402)**	
	
	**< 65 yrs. (40%)**	**> 65 yrs. (60%)**	**< 65 yrs.** **(54%)**	**> 65 yrs.** **(46%)**	**< 65 yrs.** **(80%)**	**> 65 yrs.** **(20%)**
	
**Gender**						
- Male	32 (30-34)	39 (37-40)	38 (34-42)	42 (38-47)	40 (38-43)	60 (55-65)
- Female	68 (66-70)	61 (60-63)	62 (58-66)	58 (53-62)	60 (57-62)	40 (35-45)
**Age** **(mean with 95% CI's)**	55.7 yr. (55.3-56.0)	76.1 yr. (75.8-76.3)	46.4 yr. (45.4-47.4)	75.1 yr. (74.6-75.6)	44.9 yr. (44.2-45.5)	73.3 yr. (72.7-73.8)
**Marital status**						
*Partner*	71 (69-73)	54 (52-56)	77 (74-81)	63 (59-66)	85 (83-87)	67 (62-73)
- Married	55 (53-57)	48 (46-49)	75 (72-78)	60 (57-65)	71 (68-73)	62 (57-68)
- Living together	10 (9-11)	3 (3-4)	0.7(0.3-1.6)	0.5 (0.2-1.4)	12 (10-14)	2 (1-5)
- Otherwise	7 (6-8)	3 (3-4)	1 (1-3)	1 (1-3)	3 (2-4)	3 (1-5)
						
*Single*	29 (27-31)	46 (44-48)	23 (19-26)	37 (34-42)	15 (13-17)	33 (27-38)
- Divorced	9 (8-11)	7 (6-8)	3 (2-4)	3 (2-4)	5 (4-7)	8 (5-11)
- Widowed	8 (7-9)	33 (32-34)	2 (1-4)	25 (21-29)	2 (2-3)	22 (17-27)
- Otherwise	12 (10-13)	6 (5-7)	17 (15-20)	10 (8-12)	7 (6-9)	3 (2-6)
**Children**						
- Children, good relation	59 (57-61)	74 (73-76)	68 (65-71)	71 (67-75)	72 (70-75)	78 (73-83)
- Children, bad relation (with some or all)	6 (5-7)	11 (10-12)	4 (3-6)	14 (11-17)	5 (4-6)	12 (8-16)
- No children	35 (33-37)	15 (14-16)	28 (25-31)	15 (13-19)	23 (20-25)	10 (7-13)
**Education**						
- Elementary or basic vocational	11 (10-12)	19 (17-20)	24 (21-27)	49 (45-53)	19 (17-22)	34 (29-40)
- Secondary	29 (27-31)	32 (30-33)	38 (35-42)	29 (25-33)	39 (37-42)	30 (25-35)
- Higher	60 (58-62)	50 (48-52)	37 (34-41)	22 (19-26)	41 (39-44)	36 (31-41)
**Life stance**						
- No belief	69 (67-71)	60 (58-62)	1 (1-2)	1 (0-2)	47 (44-50)	24 (20-30)
- Roman Catholic	11 (10-13)	10 (9-11)	6 (4-8)	11 (8-13)	26 (24-29)	42 (36-48)
- Protestant	6 (5-7)	14 (12-15)	92 (90-94)	87 (84-90)	21 (19-24)	27 (22-33)
- Humanistic	8 (7-9)	11 (10-12)	0 (0-0.4)	0 (0-0.4)	2 (1-3)	4 (2-6)
- Other	6 (5-7)	5 (4-6)	1 (0-1)	1 (1-3)	3 (2-5)	3 (2-6)
**Belief and its importance in someone's life**						
- Belief important	15 (14-17)	23 (21-24)	97 (96-98)	98 (96-99)	28 (25-31)	55 (49-60)
- Belief not important or no belief	85 (83-86)	77 (76-79)	3 (2-4)	2 (1-4)	72 (69-75)	45 (40-51)
**3 Characteristics from NEO-FFI** (mean scores with 95% CI's)*						
- Neuroticism	28.7 (28.3-29.0)	28.9 (28.6-29.1)	30.1 (29.5-30.7)	30.0 (29.3-30.6)	29.0 (28,6-29.4)	29.1 (28.3-29.9)
- Altruism	45.2 (44.9-45.4)	44.5 (44.3-44.7)	46.3 (45.9-46.7)	45.9 (45.5-46.3)	44.9 (44.6-45.2)	43.9 (43.4-44.5)
- Conscientiousness	46.1 (45.8-46.3)	44.8 (44.6-44.9)	45.8 (45.4-46.3)	45.0 (44.5-45.5)	45.5 (45.3-45.8)	44.7 (44.1-45.3)
**Experienced health**						
- (Very) good	80 (78-81)	74 (73-76)	86 (83-89)	77 (73-80)	89 (87-90)	79 (74-83)
- Less than good	20 (19-22)	26 (24-27)	14 (11-17)	23 (20-27)	11 (9-13)	21 (17-26)
**Suffering from a disease**						
- Yes	49.5 (47-52)	68 (66-69)	39 (36-43)	65 (61-69)	33 (31-36)	61 (55-66)
- No	50.5 (48-53)	32 (31-34)	61 (57-65)	35 (31-39)	67 (64-69)	39 (34-45)
**Resuscitation in case of cardiac arrest** **when suffering from advanced stage cancer**						
- (Probably) yes	2 (1-3)	4 (3-4)	47 (43-51)	46 (42-51)	12 (10-14)	20 (16-25)
- (Probably) no	98 (97-99)	96 (96-97)	53 (49-57)	54 (49-58)	88 (86-90)	80 (75-84)

* The Neurotics-Extroversion-Openness Five Factor Inventory (NEO-FFI), a questionnaire to test personality, has normally 5 characteristics. We only used 3, because these were the ones most relevant for our research. A respondent who filled in the questionnaire got a score on each characteristic that could range from 12 to 60; 12 meaning they scored very low on that specific characteristic and 60 that they scored very high. We took the mean score per respondent group.

NVVE-respondents are more often single, higher educated and non-religious. The majority of NPV-respondents are supporters of the protestant belief, compared to approximately one quarter of the Dutch public. The older respondents on average have a lower education.

When it comes to more specific characteristics it is clear that the younger respondents from the NVVE have more health problems compared to the Consumers' panel. Regarding a case that was presented to the respondents about preferences when suffering from advanced stage cancer, a significantly larger part of the NPV-respondents stated they wanted to be resuscitated in case of a cardiac arrest compared to the Dutch Public.

From the three characteristics from the NEO-FFI the mean scores are presented. A person who completes the questionnaire gets a score on each of these three characteristics ranging from 12 (low) and 60 (high). As can be seen in table 1, the mean scores scarcely differ between the different groups.

### Analysis

From the comparison on background characteristics between the three groups described above follows that the data originating from the NPV and NVVE cannot be merged, but rather has to be analyzed separately, because the differences between these two groups are so substantial.

Descriptive and multivariate regression analyses will be used to determine associations between patient characteristics, motivations and expectations of people. Longitudinal data analysis will be used to determine changes in time within a person regarding preferences.

Qualitative research data analysis starts during the data-collection. In this way, the already gathered data will shape the ongoing data collection in order to refine questions and develop and test hypotheses. These types of analyses are called sequential analyses.

## Discussion

The response in the first round was fairly low with 43% of the NVVE-members and 42% of the NPV-members who were approached responding. There were some factors that may explain these rates. The membership files with information about addresses from both associations were somewhat outdated. Sometimes the ADs were requested quite some time before and moving house was not always reported. With respect to the NVVE, members often requested an AD when they were already seriously ill. This made that they were either not in the mental or physical state to be able to respond to an invitation to participate in a study or they were possibly committed to a hospital or other care establishment which compromised a response. Members of the NPV were on average younger as compared to their counterparts from the NVVE, which possibly made that they were less occupied with issues concerning the end of life. While the response in the first round was low, the response in the follow-up was high for both groups, which is a fact of importance when it comes to a cohort study.

A limitation of this study is that the two groups in our cohort are not entirely representative for the part of the Dutch population who possesses an AD, because it leaves out persons who do not own a standard AD, but did draw one up for themselves. Self-written ADs are legally binding in the Netherlands as well. Nevertheless, standard ADs make up for the majority of used ADs in the Netherlands (65%) [10]. Moreover, recruiting people through the two associations, the NVVE and NPV, made that we were able to approach and include a large number of persons who actually possess an AD. Participants in the cohort are generally people who put more profound thought and contemplation into end-of-life issues and the drawing-up of an AD, which enhances the quality of the data. Another advantage is that we were able to design a prospective study, which is a great strength. Although it is not necessary in order to answer the main research questions, we did assess how our population, people who own an AD, was related to the general Dutch public. This gives valuable information, especially when the cohort is used in order to study end-of-life topics not directly related to ADs.

The size and design make that this study stands out. Most research on this subject was done in the US, where the knowledge about ADs is promoted by the Patient Self Determination Act what makes the American situation concerning ADs exceptional compared to the rest of the world. The studies performed in the US vary in size and quality, among which the SUPPORT study stands out because of its size and design [7,11-16]. Outside of the US, studies on this subject were in general smaller and not longitudinal [17-19].

This study has a broad scope focusing on the whole course of decision-making involving ADs from formulation, possible changes along the way and the actual use at the end of life. Besides this, the possibility to compare our data with other studies with related subjects also adds to the value of this study. With this study we hope to find some answers to long-lasting questions surrounding a broad scale of subjects concerning ADs.

## Competing interests

The authors declare that they have no competing interests.

## Authors' contributions

MW analysed and interpreted the data and drafted most of the manuscript. MR contributed to the design, acquisition of the data, analysis and interpretation of the data and revised the manuscript critically for important intellectual content. HP also contributed to the design, acquisition of the data, analysis and interpretation of the data and revised the manuscript critically for important intellectual content. PK contributed to the analysis and interpretation of the data and revised the manuscript critically for important intellectual content. BO contributed to the design, acquisition of the data, analysis and interpretation of the data and helped drafting parts of the manuscript.

All authors have given final approval of the version to be published.

## Pre-publication history

The pre-publication history for this paper can be accessed here:

http://www.biomedcentral.com/1471-2458/10/166/prepub
